# Analytical and Experimental Performance Analysis of Enhanced Wake-Up Receivers Based on Low-Power Base-Band Amplifiers

**DOI:** 10.3390/s22062169

**Published:** 2022-03-10

**Authors:** Lydia Schott, Robert Fromm, Ghada Bouattour, Olfa Kanoun, Faouzi Derbel

**Affiliations:** 1 Smart Diagnostic and Online Monitoring, Leipzig University of Applied Sciences, Wächterstrasse 13, 04107 Leipzig, Germany; robert.fromm@htwk-leipzig.de (R.F.); faouzi.derbel@htwk-leipzig.de (F.D.); 2 Measurement and Sensor Technology, Chemnitz University of Technology, Reichenhainer Straße 70, 09126 Chemnitz, Germany; ghada.bouattour@etit.tu-chemnitz.de (G.B.); olfa.kanoun@etit.tu-chemnitz.de (O.K.)

**Keywords:** wake-up receiver (WuRx), operational amplifier, transistor, ultra low-power design, passive RF architecture, envelope detector, Schottky diode, performance

## Abstract

With the introduction of Internet of Things (IoT) technology in several sectors, wireless, reliable, and energy-saving communication in distributed sensor networks are more important than ever. Thereby, wake-up technologies are becoming increasingly important as they significantly contribute to reducing the energy consumption of wireless sensor nodes. In an indoor environment, the use of wireless sensors, in general, is more challenging due to signal fading and reflections and needs, therefore, to be critically investigated. This paper discusses the performance analysis of wake-up receiver (WuRx) architectures based on two low frequency (LF) amplifier approaches with regard to sensitivity, power consumption, and package error rate (PER). Factors that affect systems were compared and analyzed by analytical modeling, simulation results, and experimental studies with both architectures. The developed WuRx operates in the 868 MHz band using on-off-keying (OOK) signals while supporting address detection to wake up only the targeted network node. By using an indoor setup, the signal strength and PER of received signal strength indicator (RSSI) in different rooms and distances were determined to build a wireless sensor network. The results show a wake-up packets (WuPts) detection probability of about 90% for an interior distance of up to 34 m.

## 1. Introduction

Wireless sensor networks (WSNs) have gained more and more interest in the recent period due to their importance in the IoT area, including wide applications, e.g., Industry 4.0, smart buildings, smart environment [1], precision farming [2], and health care [3]. Due to the availability of small, inexpensive, and smart sensors [4], wireless sensor networks have emerged as one of the most promising technologies of the future [5]. However, a number of challenges must be overcome to facilitate the wider deployment of WSN technologies in real-world settings, e.g., limited energy budget and processing capabilities, dynamic environmental conditions, interference and collisions in radio communications, and susceptibility to node failures [6]. WSNs are usually powered by batteries with limited lifetime. Depending on system applications, energy requirements, and availability of energy sources [7,8], other solutions, e.g., energy transfer [9] or energy harvesting [10], could be mentioned in order to ensure a continuous power supply. On the other hand, to reduce the WSN power consumption, investigation of the hardware design of nodes and the optimization of the network regarding the communication such as modulation [11], the data rate [12], network protocol [13], and clustering [14] are needed.

**Figure 1:**
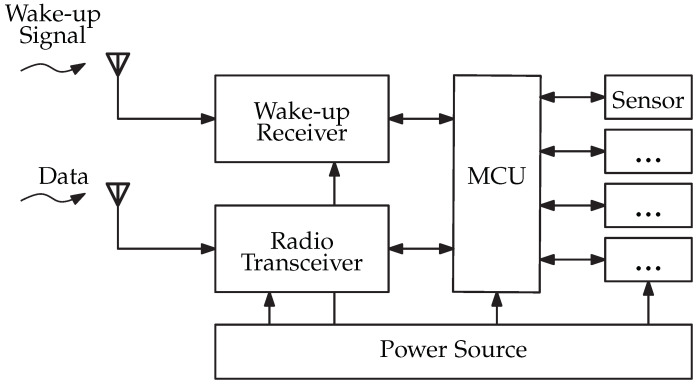
Block diagram of sensor node’s functional units.

The communication between sensor nodes can be purely synchronous [15], purely asynchronous [16], or pseudo-asynchronous [17]. Purely synchronous communication aims to establish a communication between the nodes in a certain time specified by a counter, which limits the flexibility of communication in real applications. Due to the clock synchronization and the clock drifts, the necessity of correction could additionally increase the power consumption. Pseudo-asynchronous communication exchanges data without the need for clock synchronization. The node is switching between the active and sleeping mode. The transmitter uses a preamble with an integrated address to communicate with the receiver according to an asynchronous schedule, where the reception readiness is confirmed with an acknowledgment. Usually, this leads to long latency. In order to optimize this approach, a wake-up receiver could be added. The wake-up receiver is always able to receive signals, so an on-demand communication could be realized, which reduces the power consumption of the WSN devices [18]. The so-called WuRx is connected to the sensor node, and the main microcontroller unit (MCU) (Figure 1) remains in the power-saving sleep mode. The WuRx listens for special telegrams called WuPts. If such a packet is received, an interrupt is generated and the MCU changes from the sleep mode to the active mode.

The typical structure of a sensor node consists of an MCU, a radio transceiver, one or more sensor elements, and a power source [19]. Sensor elements acquire data on the environmental conditions, including the processing unit, to handle and forward information wirelessly to other sensor nodes or a base station via the radio transceiver. For WuRx reception, a second antenna is used. The WuRx is connected to the MCU for configuration and wake-up signaling.

In this paper, the performance and reliability of sensor nodes with integrated wake-up receivers based on commercial of the shelf (COTS) components are investigated in an indoor environment. The goal is to achieve a very low PER up to the sensitivity limit in the sub-10 μW range. Therefore, two different LF amplifier are introduced to increase the WuRx sensitivity. The focus is on the reliable reception of the WuPts in view of obstacles in the interior, such as masonry and furniture. Sensitivity, range, data rate, and power requirements are taken into account.

The structure of this paper is as follows: In Section 2, the approach is compared and discussed with techniques from State of the Art (SoA). Based on passive and low-power components, a WuRx architecture is built. Section 3 describes the theoretical investigation and simulation of the individual components of the structure with respect to the application of a wireless sensor node in the 868 MHz band. Subsequently, the results are verified with experiments in the laboratory and under real test conditions. The experimental setup and measurements are presented in Section 4. Finally, all results are discussed in Section 5.

## 2. Related Works

A common mechanism for reducing power consumption in listening mode is duty-cycling. When duty-cycled, devices wake up at selected times, listen for defined time slots, and go back to a sleep mode; hence, the battery lifetime could be extended. Unfortunately, the planned duty-cycling technique is always a trade-off between energy consumption, sensitivity, and a higher mean communication latency [20]. Since the WuRx is usually in listening mode for a long time period, the selection of the circuit elements of WuRx is very critical, especially for high-frequency signals where the energy consumption, the sensitivity, and the reliability of the received packets are significantly challenging, especially with regard to their interactions between each other. The WuRx hardware is generally based on special electronic circuits designed to reduce power consumption without reduction in sensitivity, reliability, and data rate. The hardware design of WuRx can be based on integrated complementary metal-oxide semiconductor (CMOS) circuits [21,22,23], where they show a complicated fabrication process that may need a longer development time period within expensive procedures, in contrast to circuits based on COTS components that are usually cheaper in setup, maintenance, expansion, and development. In order to save required energy in listening mode, the WuRx based on COTS uses generally passive components for the continuous listening to the radio channel to activate the MCU [24].

Various WuRx circuit architectures have been developed to reach a compromise between the sensitivity and data rate, as well as power consumption. Analyzing the data communication and wake-up functionalities into one WuRx hardware was presented in [25]. The latency, the current consumption, and the overall operation range performance under different transmit power levels were characterized. The authors developed a WuRx without an LF amplifier. The system was evaluated in an outdoor setup with a performance range up to 40 m by a transmission power of 11 dBm. The presented approach of WuRx without any amplification stage and no RF bandpass filter can affect the sensitivity and reliability, where interferences from other RF devices can affect the system dramatically.

Ref. [26] presents a WuRx with a comparator situated between envelope detector and low-power microcontroller to sense smaller input signals and generate the interrupt to the MCU. A wide range of sensitivity with the received packets sequences has been tested in the case of two working frequencies, 433 MHz and 868 MHz. Results present experiments evaluated within the same region of the nodes up to 24 m, a packet receiving sequence of 99% and 96% for the outdoor and indoor scenarios, respectively. The proposed WuRx achieved a sensitivity of −55 dBm, but the data rate is not mentioned.

An approach to enhance the sensitivity focuses on the use of voltage multiplier to collect the weak RF energy from the antenna. Proposed in [27], the envelope detector circuit with two or more voltage stages acts as a charge pump. However, increasing the number of voltage stages will reduce the whole efficiency of the envelope detector. The voltage at the input decreases because of smaller input resistance of envelope detector and higher conductance [28].

Another technique is the use of an RF amplifier, or low noise amplifier (LNA) situated between antenna and detection circuit. The authors in [29] propose an LNA circuit that consists of a commercial component with a low shutdown current, short turn-on time, and high amplification. The developed circuit reaches a sensitivity up to −71 dBm, but with a current requirement of 5.8 mA when permanently active. A data rate of 250 kbit/s and an average power consumption of 8.1 μA can be achieved. Due to the duty-cycling, the transmitter has to be active until WuPt is received from WuRx. On the other hand, this affects the latency, reliability, and current consumption of the system. The authors in [30] used a two-stage LNA based on bipolar junction transistors (BJTs) and achieved a sensitivity of −90 dBm. However, the presented architecture means a higher circuitry effort due to numerous passive components. The average power consumption comes to 3 μW and data rate is 128 kbit/s. The higher data rate is necessary to reach the listening time of the WuRx when using during duty-cycling protocol. Furthermore, the extended active time of transmitter increases the current consumption.

In [28], the authors used an additional amplifier stage situated between detection circuit and interrupt generator. The developed circuit with LF-amplification stage based on OAs achieves a sensitivity of −55 dBm with a power requirement of 1.2 μW. The study does not mention the data rate. In [31], a higher sensitivity of −70 dBm is gained with this approach. The signal is amplified with an OA, and the comparator is used as preamble detector, using an additional microcontroller for address-matching of the WuPt and triggering the MCU in order to process the signal. The high sensitivity is achieved but only for a very low data rate of around 40 bit/s. Compared to data rate in the range of 1 kbit/s, the latency and current requirement increases by a factor of 25.

On the other hand, in [32,33], the research study shows a WuRx circuit with LF-amplifier made from two high-gain BJTs. The results provide high sensitivity with an input power of −63 dBm to supply a WuRx with a power of 8.7 μW and 7.4 μW. It has to be noted that these designs are working in the 433 MHz band. A slight increase of sensitivity compared to 868 MHz can be expected. The presented circuits show a high sensitivity due to the integration of a BJT amplifier based on discrete components integrated between the enveloped detector and the interrupt generator. The authors investigate proposed WuRx circuit performances for wireless sensor networks under laboratory conditions. A reliability study is not provided.

It should be noticed that sensitivity, power consumption, and data rate are always a trade-off in terms of performance of the WuRx architecture. Table 1 summarizes the mentioned properties of the approaches from the SoA.

**Figure 2:**
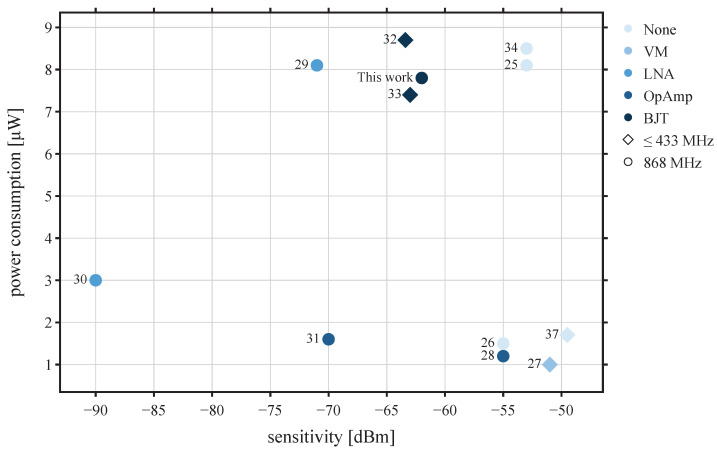
Comparison of SoA WuRx architectures.

Figure 2 shows an overview of the described WuRx architectures based on their power consumption, sensitivity, carrier frequency, and LF amplifiers. It can be seen that WuRx with RF amplifiers [29,30] achieves sensitivity better than −70 dBm with a power consumption of 8 μW. However, the integration of the LNA increases the average current consumption and the latency of the system compared to an always-on listening approach. On the other hand, WuRx architectures with voltage or LF amplifier circuit [26,27,28] are considered as low power consumption WuRx of less than 2 μW, where their sensitivity is considered low, in the range of only −55 dBm. This suggests a correlation between sensitivity, data rate, and power requirement. Thus, there are trade-offs to be made when designing WuRx architectures. The use of amplifiers in LF range is important to enhance the performance of WuRx without increasing power consumption and decreasing sensitivity and data rate. It can be seen that the architecture based on transistor and operational amplifier show a high performance on low power consumption and high sensitivity, respectively.

Based on the SoA, the amplifiers with OAs and transistors are used to reach a sensitivity of more than −50 dBm within a power consumption lower than 10 μW. Compared to other works based on LF amplifier approach, a higher sensitivity of −62 dBm is reached with the frequency range 868 MHz. For that, an analytically and experimental analysis and comparison between both architectures in terms of simple hardware design, low power requirement, and high data rate is proposed. Furthermore, to determine reliability, the relationship between sensitivity and number of WuPts received is examined.

Their analysis will be within the proposed application, and an indoor setup is built to test the performance of both architectures, which may be of interest for building a wireless sensor network across different rooms and floors. Since the implementation of LF-amplifiers in WuRx circuits represents a very interesting compromise between sensitivity, data rate, latency, and low power consumption, further investigations are required in order to find out the best operating mode.

## 3. Analytical Expressions

The different parts of WuRx are investigated analytically as well as with simulations. These will include the power consumption from each stage and operating mode.

### 3.1. General Structure of the Adopted WuRx Circuit

**Figure 3:**
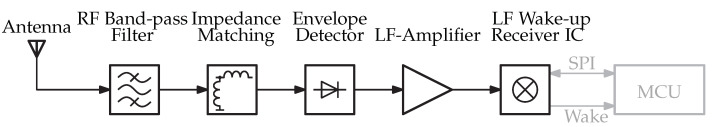
Block diagram of the WuRx architecture.

The general structure of a WuRx is shown in Figure 3, which is composed of antenna, RF bandpass filter, impedance matching, envelope detector, LF-amplifier, and LF wake-up receiver IC.

To reduce interference with other radios and to avoid disturbances from different frequency bands, a bandpass filter is used to let the input signals of a certain frequency range pass. The Schottky diodes after the filter demodulate the incoming wake-up signal. For encoding the WuPt, a low-power microcontroller [34,35], flip-flops [36], or preamble detector [37] are employed. To identify the intended received signal and prevent false wake-ups, the WuPt can contain an address field with a node ID. After address matching, the decoder generates an interrupt to the main MCU.

As shown in Figure 3, the architectures of OA and BJT as LF-amplifier approach consist of a bandpass filter for 868 MHz, a matching network, the detector diodes, and the low-power LF wake-up receiver IC for address matching of the wake-up signal. After the detector diodes, the LF signal is amplified by an LF-amplifier circuit. To identify the right signal, an address decoder is used. Usually, the AS3933 is employed as a decoder and is able to detect signals with a minimum voltage level of at least 80 μVRMS. This means that the AS3933 limits the overall sensitivity of the WuRx. For this reason, amplification has to be implemented in order to increase the sensitivity of the whole receiver to the minimum detectable voltage level. The AS3933 chip analyses the wake-up signal in order to check the validity of the message and the matching to the predefined address. In case of validation and matching, the MCU is woken up by an interrupt of the LF wake-up receiver chip.

The main differences between these both architectures are concerning the amplification stage. The first architecture uses a transistor-based amplifier (named as BJT) and the second one uses operational amplifier in a transimpedance amplifier configuration (named as TIA).

### 3.2. Energy Consumption

**Figure 4:**
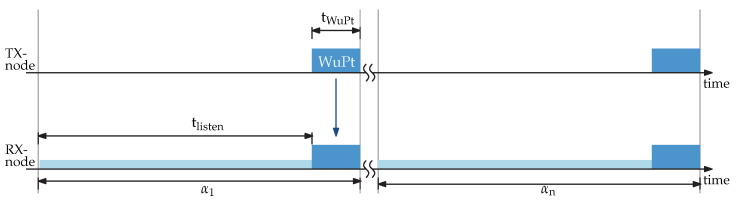
Signal timings of wake-up intervals.

Both architectures are based on always-on technique. Figure 4 displays a simplified timing diagram of channel listening and WuPt. The transmission node (TX-node) is sending a packet and the WuRx (RX-node) is continuously listening to the channel for the WuPt. After data receiving, the LF wake-up receiver chip is matching the address before sending the interrupt to the MCU.

During a wake-up interval α, the time of channel listing $t_{\mathrm{listen}}$ the energy consumption of listening mode $E_{\mathrm{listen}}$ can be calculated as shown in Equation (1).

(1) $$E_{\mathrm{listen}}=\left(P_{\mathrm{AS}3933\_\mathrm{listen}}+P_{\mathrm{LF}\_\mathrm{Amp}}+P_{\mathrm{MCU}\_\mathrm{LPM}}+P_{\mathrm{RF}\_\mathrm{LPM}}\right)\;\cdot \;t_{\mathrm{listen}}$$

where $P_{\mathrm{AS}3933\_\mathrm{listen}}$, $P_{\mathrm{MCU}\_\mathrm{LPM}}$, and $P_{\mathrm{RF}\_\mathrm{LPM}}$ are the power consumption of low-power mode during listening mode of the LF wake-up receiver chip, microcontroller, and RF transceiver, respectively. $P_{\mathrm{LF}\_\mathrm{Amp}}$ is the active current of the LF amplifier.

(2) $$E_{\mathrm{RX}\_\mathrm{WuPt}}=\left(P_{\mathrm{AS}3933}+P_{\mathrm{LF}\_\mathrm{Amp}}+P_{\mathrm{MCU}\_\mathrm{LPM}}+P_{\mathrm{RF}\_\mathrm{LPM}}\right)\;\cdot \;t_{\mathrm{WuPt}}$$

$E_{\mathrm{RX}\_\mathrm{WuPt}}$ represents the energy required for WuPt reception, as expressed in Equation (2). The supply power of BJT or transimpedance amplifier (TIA) is written as $P_{\mathrm{LF}\_\mathrm{Amp}}$ and $P_{\mathrm{AS}3933}$ is the power of AS3933 in detecting mode. The energy of receiving the WuPt is determined by $t_{\mathrm{WuPt}}$. The energy requirement during sending the WuPt is displayed in Equation (3) with a transmission power of the transceiver $P_{\mathrm{RF}}$.

(3) $$E_{\mathrm{TX}\_\mathrm{WuPt}}=\left(P_{\mathrm{MCU}}+P_{\mathrm{RF}}\right)\;\cdot \;t_{\mathrm{WuPt}}$$

### 3.3. Path and Antenna Losses

The frequency band of 868 MHz is used as the radio channel for the wake-up signal. This frequency band is regulated in terms of transmission power and duty cycle and often used for many application, e.g., building automation. The use of high frequencies for the wake-up signal leads to high power consumption in the receiver due to switching losses. On the other hand, the use of low frequencies leads to larger antenna and lower gain [34,36]. The amplitude shift keying (ASK) is known for its simplicity to modulate the carrier signal and create the WuPt sequence pattern. By switching the carrier frequency on and off—OOK—the pattern is generated. Therefore, the OOK modulation enables a simple and energy-efficient hardware design for demodulation, e.g., by using an envelope detector consisting only of diodes. Here, the frequency and phase remain unchanged and only the amplitude varies to represent the two binary values. When the WuPt is received, the envelope detector demodulates the high-frequency carrier signal to obtain a low-frequency wake-up signal [38]. A disadvantage of ASK modulation is that noise and interference affect the amplitude. Another modulation option for wake-up receivers is frequency shift keying (FSK), as it is less susceptible to noise compared to ASK. However, the FSK circuit consumes more power because a frequency synthesizer is needed to distinguish between the central frequency and the frequency deviation.

In indoor applications, interferences, as well reflections, diffraction, and attenuation, are expected and should be taken into consideration. For that purpose, many research activities focused on the development of path loss models to predict the propagation of electromagnetic waves. The basic path loss model is the free space model (Equation (4)) [39], which predicts an inverse square dependence of the average received power on the distance between the transmitter and receiver.

(4) $$\frac{P_{r}}{P_{t}}=G_{t}\;\cdot \;G_{r}\;\cdot \;{\left(\frac{\lambda }{4\pi d}\right)}^{2}$$

where $P_{r}$ and $P_{t}$ are the received and transmitted signal powers, $G_{r}$ and $G_{t}$ are the gains of the antennas, *d* is the distance between antennas, and λ is the wavelength. Modified models attempt to account for the complex nature of real wireless channels.

**Figure 5:**
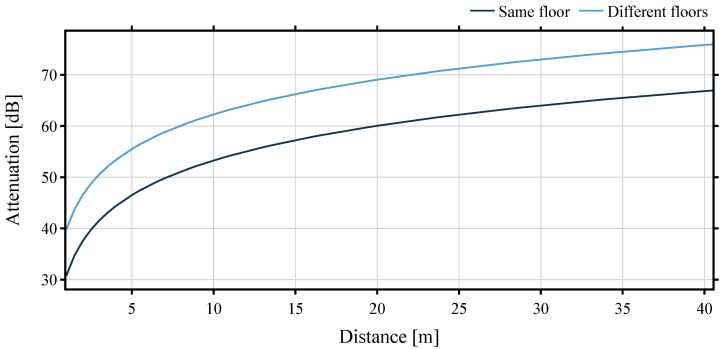
Theoretical attenuation over a distance up to 40 m.

The International Telecommunication Union (ITU) model for indoor attenuation considers loss through multiple floors, as well as transmission through walls. The coefficients from [40] implicitly take into account obstacles and other loss mechanisms that may occur inside a floor in buildings. The basic model is given by

(5) $$L_{\mathrm{total}}\left[\mathrm{dB}\right]=L\left(d_{0}\right)+N\log_{10}\frac{d}{d_{0}}+L_{f}\left(n\right)$$

where *N* is the distance power loss coefficient, $L_{f}$ the floor penetration loss factor, $L\left(d_{0}\right)=20\mathrm{dB}\;\cdot \;\log_{10}f-28\mathrm{dB}$ is basic transmission loss at reference distance $d_{0}$ and carrier frequency *f* in MHz and assuming free-space propagation. Figure 5 shows the losses for a distance from up to 40 m across one floor and through walls.

### 3.4. Impedance Matching and Envelope Detector

An impedance matching network is required to maximize the received RF power of the antenna. To ensure the best possible performance of the diode circuit, the load should be equal to the conjugate of the source impedance. The Schottky diode SMS7630 from Skyworks was chosen as the detector diode due to its sensitivity to the lowest RF power. The most common measure of mismatch is return loss $S_{11}$. To create an effective impedance matching, passive elements such as capacitors and inductors are used, which reduce the losses due to the reflection of a certain part of the energy of the incident waves. The antenna and the SAW filter are designed for 50 Ω source impedance. For the defined transmission frequency of 868 MHz, the load impedance $Z_{L}$ is defined as shown in Equation (6).

(6) $$Z_{L}=50\Omega \;\cdot \;\frac{1+S_{11}}{1-S_{11}}.$$

**Figure 6:**
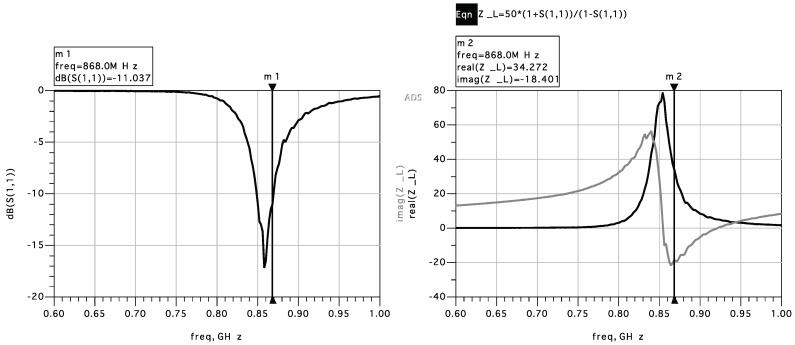
ADS simulation of reflection coefficient S11.

The reflection coefficient is simulated in ADS Keysight (Figure 6) and the SPICE parameters of the SMS7630 diode. It is found that the diodes are almost entirely capacitive and are matched with two inductors to achieve the best resonant frequency.

**Figure 7:**
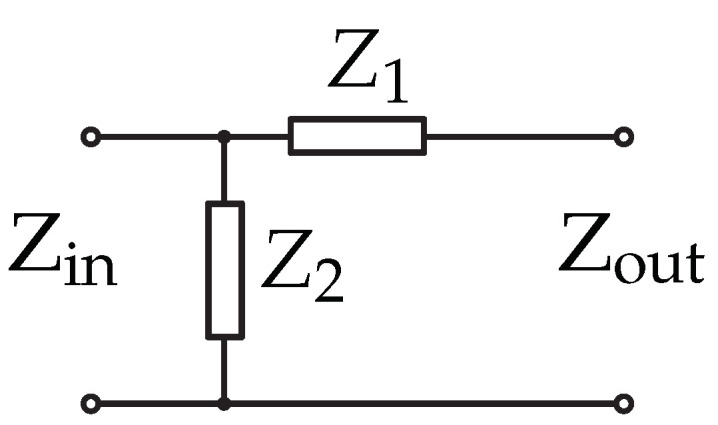
Reversed L network [41].

The matching network evaluated by ADS consists of a reversed L network (Figure 7), L${}_{1}$ = 22 nH and a parallel inductance L${}_{2}$ = 3.3 nH. The exact values of the matching network are determined directly on the board using the input reflection coefficient $S_{11}$ measurement on the network analyzer.

The maximum diode RF voltage sensitivity at room temperature at 868 MHz, assuming ideal impedance matching, is derived from [42], which is expressed in Equation (7).

(7) $$\gamma_{\mathrm{RF}}=\beta_{i}\;\cdot \;R_{V}.$$

The current sensitivity can be described in Equation (8), which is defined by the angular frequency ω, junction capacitance $C_{j0}$, and video resistance $R_{V}≃nV_{T}/I_{s}$ with saturation current $I_{s}$, and the thermal voltage $V_{T}$, as well as the series resistance $R_{s}$ and the ideality factor *n*.

(8) $$\beta_{i}=\frac{1}{2nV_{T}\left(1+{\left(\omega C_{\mathrm{jo}}\right)}^{2}R_{V}R_{S}\right)}.$$

Furthermore, the envelope detector diodes influence the WuRx sensitivity. For that, the tangential signal sensitivity (TSS), expressed in Equation (9), is used to describe the sensitivity of the detector diodes and is the lowest input signal power level [43] where $B_{V}$ is the bandwidth, *T* expresses the environmental temperature, and *k* is the Boltzmann constant.

(9) $$P_{\mathrm{TSS}}=\frac{2.5\sqrt{4kTR_{V}B_{V}}}{\gamma_{\mathrm{RF}}}$$

**Figure 8:**
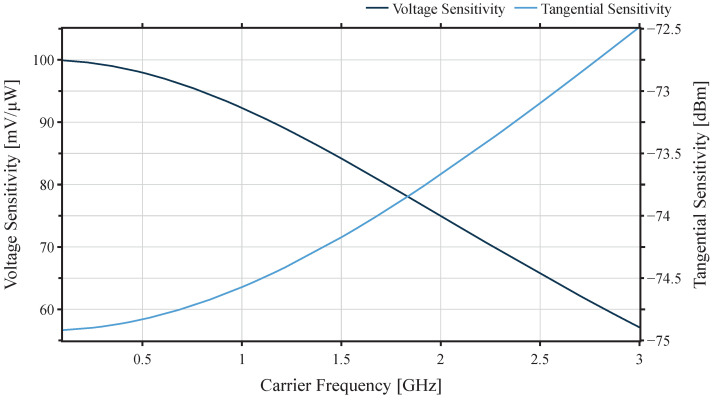
Voltage sensitivity and tangential sensitivity depending on signal frequency.

The curve of voltage sensitivity and tangential sensitivity are shown in Figure 8. With T=298K and $B_{V}=18.7\mathrm{kHz}$ at 868 MHz, the voltage sensitivity is more than 90 mV/μW; according to this value, the lowest input power level of the diodes is −74.7 dBm.

**Figure 9:**
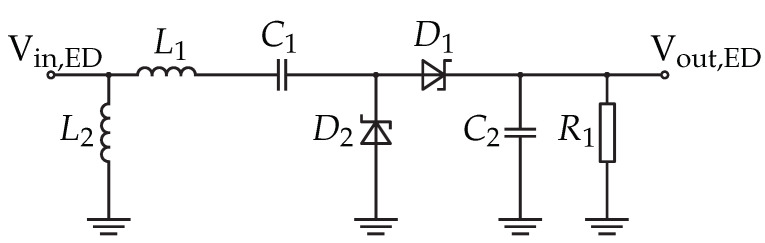
Schematic of the envelope detector.

The envelope detector circuit is designed using Greinacher voltage multipliers as given in Figure 9. It is used to demodulate the received wake-up signal, as well as the amplification of the input voltage ($V_{\mathrm{in}},\mathrm{ED}$). Typically, the output voltage of the voltage multiplier ($V_{\mathrm{out}},\mathrm{ED}$) is almost the double of the input voltage, where the used ones affect the voltage level by their reverse voltage.

### 3.5. Low-Frequency Amplifiers

The amplification of the signal after the detector circuit is important to reach the minimum detectable voltage level from AS3933 of $V_{\mathrm{AS}3933,\mathrm{RMS}}=80\;µV$ [44]. With γ=40 mV/µW, a peak-to-peak value, output voltage of the envelope detector of $V_{\mathrm{ED},\mathrm{PP}}=12\;µV$ at −65 dBm, can be approximated. Equation (10) shows the calculation of the required voltage gain *G*.

(10) $$G=\frac{V_{\mathrm{AS}3933,\mathrm{RMS}}}{V_{\mathrm{ED},\mathrm{RMS}}}=2\sqrt{2}\;\frac{V_{\mathrm{AS}3933,\mathrm{RMS}}}{V_{\mathrm{ED},\mathrm{PP}}}=17.9$$

These amplification stages can be implemented by different circuits. As can be seen from Figure 2 OAs and BJT, amplifiers are the most common. Both architectures shall be investigated in the following subsections.

#### 3.5.1. Transimpedance Amplifier (TIA)

**Figure 10:**
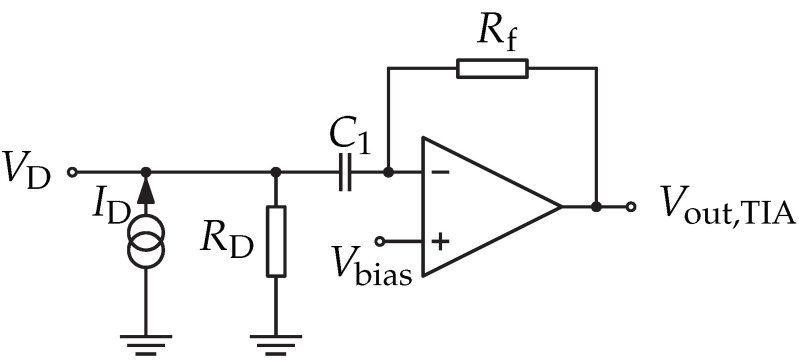
Schematic of the TIA amplifier.

The envelope detector circuit can be modeled as a Thévenin equivalent with an internal current source that can be easily useful for the modeling with other WuRx stages. The TIA circuit acts similar to a current-to-voltage amplifier with an adjustable conversion factor. TIA circuits are typically implemented as OA-based amplifiers. Figure 10 shows the schematic used in this implementation. The TIA is supplied by $V_{V}+=3.0V$.

The TIA output voltage is defined as shown in Equation (11), which depends on $R_{D}$ the diode’s Thévenin resistance, $I_{D}$ the diode’s source current, and $R_{f}$ the TIA’s feedback resistor.

(11) $$V_{\mathrm{out},\mathrm{TIA}}=-R_{f}\;\cdot \;I_{D}=-\frac{R_{f}}{R_{D}}\;\cdot \;U_{d}$$

With $R_{f}$ = 200 kΩ and $R_{D}\approx$ 10 kΩ, the required voltage gain of 20 was reached. A reference voltage of 1.2 V was used to ensure that the proper output voltage swings. $C_{1}$ is the corresponding bias-blocking capacitor interrupting the DC current flow towards the envelope detector.

The performance of the TIA is mainly related to the used OA where the supply current and gain bandwidth product (GBWP) are the main challenging elements during their selection within an application. In fact, the current requirement of the OA should be as small as possible, resulting in a low GBWP value.

#### 3.5.2. BJT-Based Amplifier Circuit

**Figure 11:**
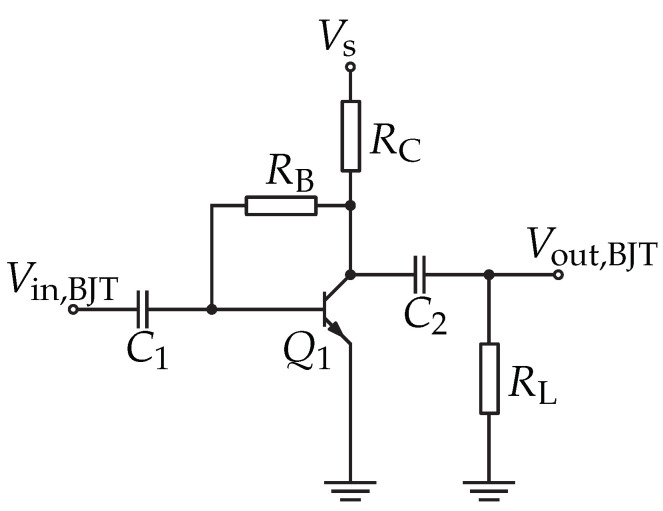
Schematic of the BJT amplifier with emitter architecture.

The bipolar transistor can be operated in three basic circuits: common emitter, common collector, or common base configuration. Because of its high voltage gain and high input impedance, the common emitter circuit was selected for a single-stage amplifier, as shown in Figure 11, where the BJT transistor is named as $Q_{1}$.

Typically, the amplifier based on BJT requires a specific operation point. These operation points can be highly influenced by temperature changes and component tolerances. These problems can be solved by connecting $R_{B}$ parallel to collector and base. This creates the stable operating point, but limits the output voltage span, due to $V_{\mathrm{CE}}\approx 0.7V$.

**Figure 12:**
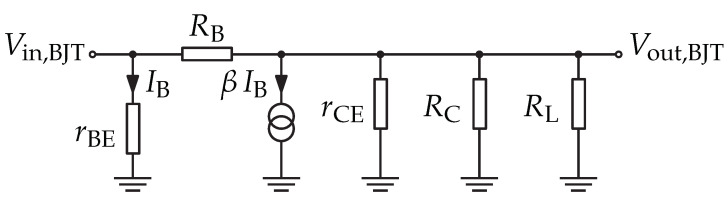
Small-signal circuit of the BJT amplifier according to [45].

A small-signal analysis according to [45] was made in order to investigate the voltage amplification. Figure 12 shows the small-signal circuit of the amplifier. Equation (12) shows the result of the small-signal analysis with $r_{\mathrm{BE}},r_{\mathrm{CE}}$, and β the small-signal parameters. Further approximations were made in order to simplify the formula. The resulting approximation is only dependent on supply voltage $V_{S}$, thermal voltage $V_{T}$, load resistance $R_{L}$, and collector resistor $R_{C}$.

(12) $$G=\frac{r_{\mathrm{CE}}\left|\right|R_{C}\left|\right|R_{L}}{r_{\mathrm{BE}}}\;\cdot \;\frac{r_{\mathrm{BE}}-\beta \;\cdot \;R_{B}}{R_{B}+(r_{\mathrm{CE}}\left|\right|R_{C}\left|\right|R_{L})}\approx -\frac{V_{S}}{V_{T}}\;\cdot \;\frac{R_{L}}{R_{C}+R_{L}}$$

Practically, for WuRx, the $R_{L}$ is the equivalent model of input resistance of AS3933.

### 3.6. Low-Frequency Wake-Up Chip

The low-frequency wake-up chip is used to generate an interrupt to the MCU with minimal influences of interferences. Typically, in real applications, the WuRx are used in environments, where interferences are expected due to the presence of other electromagnetic waves. This aspect influences the working behavior of the WuRx and leads to false wake-up events. To avoid false positives, one method based on the pattern correlation can be used to activate and to match incoming signal with valid pattern stored in the wake-up chip. The LF WuRx chip AS3933 is a low-power, three-channel ASK receiver that can generate a wake-up call to the MCU. The AS3933 is designed for carrier frequencies of 15 kHz to 150 kHz using OOK. This is not sufficient for radio transmission in the sub-gigahertz band. Therefore, the OOK signal is digitally modulated to a sub-carrier of 18.7 kHz and up-converted to 868 MHz. When the signal is detected at the receiver part, the passive envelope detector converts it back to the kHz band. It is then captured by the AS3933. For the use of the internal crystal oscillator as reference clock, the signal frequency can be chosen with Equation (13).

(13) $$f_{\mathrm{carr}}=f_{\mathrm{RC}}\;\cdot \;\frac{8}{14}$$

Based on the specification of AS3933, incoming wake-up packets must be Manchester-coded [44]. If the wake-up protocol is valid, the AS3933 will generate an interrupt to the MCU.

**Figure 13:**
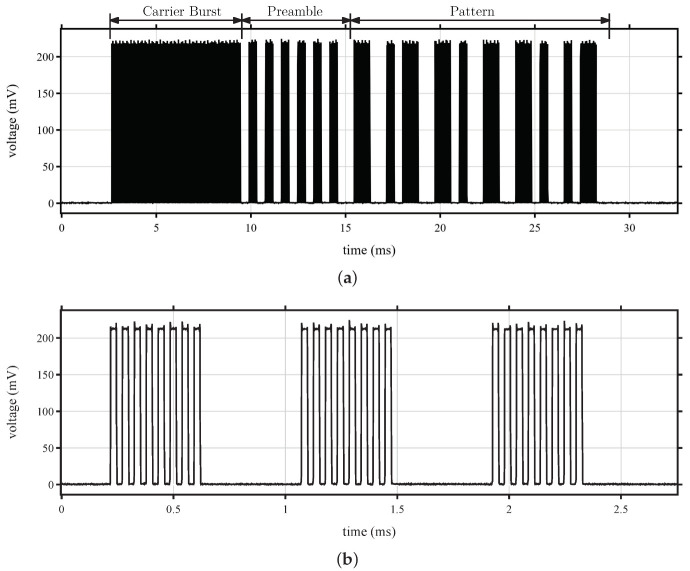
Manchester coded wake-up packet. (**a**) Pattern with carrier burst, preamble, and address; (**b**) OOK signal on kilohertz band.

Figure 13 shows the demodulated WuPt used throughout this work. The packet with enabled pattern detection is displayed in Figure 13a and consists of a carrier burst, preamble, and 16-bit address, whereas Figure 13b exposes the amplitude modulation of the signal.

For each active channel, the AS3933 provides a RSSI value. The RSSI indicates how strong the input signal is and the inverse representation of the gain of the variable gain amplifier (VGA). The gain of all channels is set to maximum in listening mode. As soon as a signal is detected, the automatic gain control (AGC) is activated and the gain of the VGA is set to the correct value and is between 0 and 31 [44]. The relationship between gain and RSSI can be shown with Equation (14) [46].

(14) $$G\;\left[\mathrm{db}\right]=G_{\mathrm{offset}}-20\mathrm{dB}\;\cdot \;\log_{10}\left(\frac{V_{\mathrm{in}}}{V_{\mathrm{ref}}}\right)$$

The offset gain of the AS3933 $G_{\mathrm{offset}}$ corresponds to 62 dB, and $V_{\mathrm{ref}}$ represents the minimum detectable power of the input signal 80 μVRMS.

## 4. Investigation Results

### 4.1. Overall System

**Figure 14:**
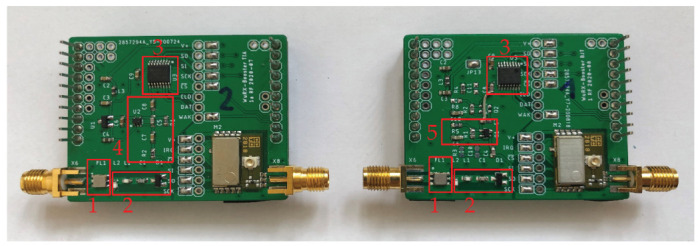
PCBs of the proposed architectures. 1—bandpass filter, 2—envelope detector, 3—LF WuRx IC, 4—TIA amplifier, 5—BJT amplifier.

In this section, the two proposed designs of the two prototypes are implemented and built on two-layer PCBs. The used BJT and TIA boards are shown in Figure 14, respectively. Their dimensions are about 40×50mm. The main characteristics and power parameters of used components are summarized in Table 2.

Both WuRx architectures are equipped with ANT-868-CW antenna and the B39871B bandpass filter to pass the signal in the 868 MHz frequency band. The envelope detector consists of two SMS7630 Schottky diodes from Skyworks Solution Inc. to demodulate the received signal. As explained in Section 3.4, the chosen diodes reach the lowest detectable RF power of −74 dBm.

The demodulated signal is amplified by the MIC861 OA at the TIA implementation or by BFP405 RF transistor at the BJT implementation. Microchip’s MIC861 OA was selected because of its GBWP of 400 kHz at a low supply current of 4.6 µA [47]. With G=20, the resulting upper corner frequency is 20 kHz, sufficient to amplify the 18.7 kHz LF signal. LTspice simulation shows that general-purpose transistors are not suitable due to a corner frequency lower than 18.7 kHz. This is coursed by high parasitic capacitance and the high $R_{C}$. The BFP405 is a high-frequency transistor, which was utilized due to its low parasitic capacitance, resulting in a corner frequency of more than 100 kHz. From Equation (12), with $V_{V}+$ = 3.0 V, $V_{T}$ = 26 mV, $R_{C}$ = 2.4 MΩ, and the AS3933 load resistance, $R_{L}$ = 2 MΩ [44] will result in G≈−58. The voltage gain is not strongly dependent on the transistor’s current gain β.

For the investigation, the WuPt transmission is generated by the SPSGRFC-868 radio module from STMicroelectronics. The MCU utilized on the boards is a 16-bit MSP430G2553 manufactured by Texas Instruments running at 8 MHz. The MSP430 can enter multiple low-power modes. In order to keep a clock crystal for timing active, the LPM3 is used. When in low-power mode 3, it consumes $P_{\mathrm{MSP}\_\mathrm{LPM}3}$ = 2.55 μW. To power the WuRx nodes, 3.0 V batteries are used.

### 4.2. Energy Consumption

The energy consumption of the WuRx mainly depends on the operating mode such as wake-up period and amount of data, as well as the used circuit elements. The length of the active time depends on the data rate and the amount of data. The data rate for the WuPt is 1160 kbit/s at a signal frequency of 18.7 kHz and with Manchester coding. The number of bits and the length of the WuPt are displayed in Table 3. Results show that the total duration of the WuPt ($t_{\mathrm{WuPt}}$) is about 27.7 ms.

**Figure 15:**
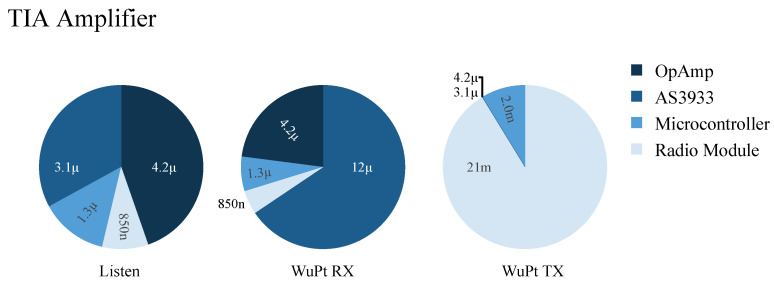
Current need of WuRx with TIA amplifier.

**Figure 16:**
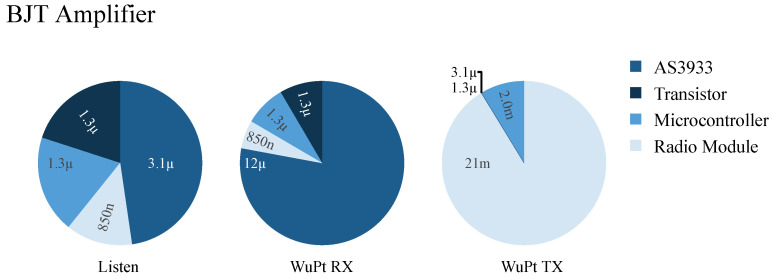
Current need of WuRx with transistor.

During the transmission of a WuPt, the energy consumption of the node depends on the active mode of the components. $P_{\mathrm{MCU}}$ and $P_{\mathrm{RF}}$ are the power consumption of microcontroller and RF transceiver during transmission mode. With $t_{\mathrm{WuPt}}$, the energy consumption is $E_{\mathrm{TX}\_\mathrm{WuPt}}$, as expressed in Equation (3). Figure 15 and Figure 16 show the power consumption of BJT and TIA according to the installed components and working mode. In listening mode, the OA of the TIA board consumes the most current compared to the other components, while for the BJT, the AS3933 has the highest amount of current consumption.

Table 4 shows the results of the current consumption of the WuRx using the BJT and the TIA amplifiers. These results have been calculated based on Equations (1)–(3) shown in Section 3.2. This includes the different working modes such as listening, receiving, and transmission for a wake-up period of 1 s. Results show that typically the listening mode is characterized with the highest energy consumption of more than 15 times compared to other working modes. Furthermore, it can be seen that the BJT amplifier leads to a lower energy consumption compared to TIA of around 1.7 μJ and 0.24 μJ in the listening and the receiving mode, respectively. In the transmission mode, both architectures consume about 1.91 mJ. This can be explained by the high current requirement of the SPSGRFC-868 radio module when sending the packets in the range of 21 mA, so that the power requirement of the amplifiers can be neglected.

**Figure 17:**
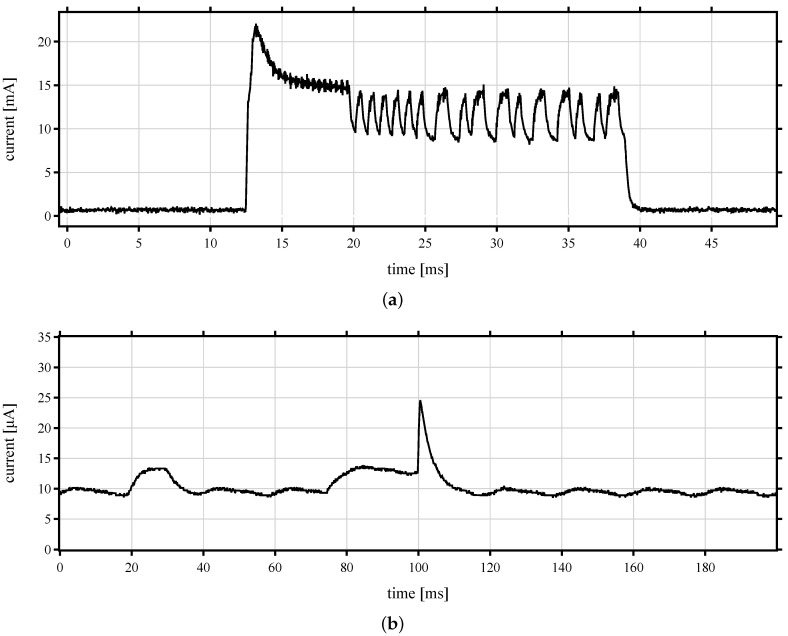

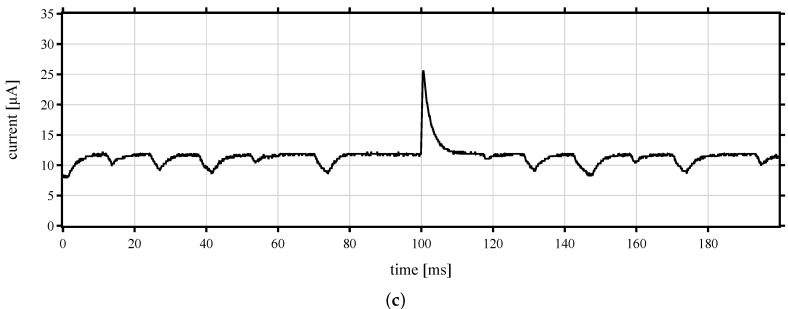
Current curve of WuPt. (**a**) Transmitter node, (**b**) receiver node with BJT, and (**c**) receiver node with TIA amplifiers.

The BJT architecture has a lower energy consumption than TIA in listening mode, whereas in active states, both PCBs require nearly the same energy. Figure 17 displays the current waveforms of the transmitting mode, receiving mode with BJT, and receiving mode with TIA, respectively.

The energy consumption reaches its maximum in the transmitting mode, where the current consumption reaches a value higher than 15 mA during 27 ms. The current consumption on the transmitter is related to transmitting power of 11 dBm of SPSGRFC-868 radio module, which is lower than the specified current consumption of the data sheet. The measured current is lower than specified in the data sheet because of ASK modulation with a duty-cycle of around 50%.

Figure 17b,c (BJT and TIA, respectively) show the signal progression at the receiver architectures. Their peak of consumption is related to the MCU, which is waking up. It can be seen also that based on Figure 17c, the signal of the TIA amplifier is very distorted, which is mainly related to AS3933. Since the LF wake-up IC is always ON and listening to the channel, the LDO is very noisy. It refers to the amount of ripple on the output coming from ripple on the input. Due to the measurement inaccuracy of the oscilloscope, the current values of Figure 17b,c are different from the calculations. Here, the effects of the RC constant are clearly visible, whereas in Figure 17a, they are not visible due to the larger measuring range in the mA range.

### 4.3. Sensitivity Measurements

**Figure 18:**
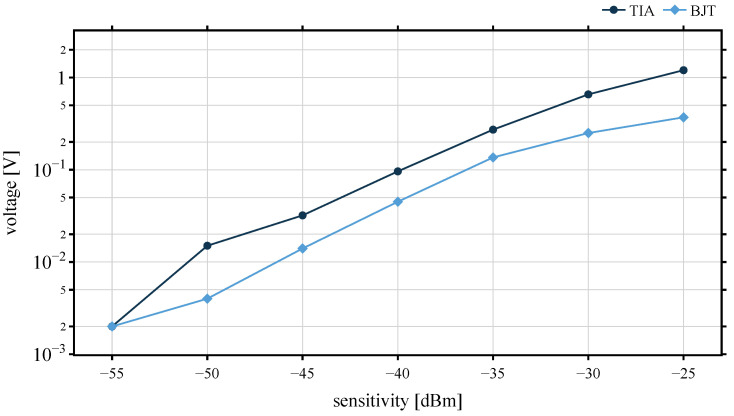
Amplification level of BJT and TIA architecture.

The minimum theoretical sensitivity of the WuRx defines the minimum detectable signal. The amplification performance of each architecture is evaluated with signal generator and an input signal with various sensitivity level. Figure 18 shows the voltage after the amplification stage of BJT and TIA board.

A poor detection mechanism will lead to packet errors, therefore the number of received WuPts versus transmitted packets is examined to study the noise immunity of the WuRx. Furthermore, the RSSI measured by the AS3933 is another value for evaluating and comparing the performance of the two architectures. The amount of transmitted packets *N* is calculated using the probability of arrival and the confidence interval and is seen in Equation (15) where *z* is the standard score, and *p* represents the probability of *e* the confidence level.

(15) $$N=\frac{z^{2}\;\cdot \;p\left(1-p\right)}{e^{2}}.$$

At a confidence level of 99%, the standard deviation corresponds to a value of 2.58. The probability was determined by a sample measurement of 1000 packets, in which all packets arrived with a probability of 90%. This leads to a sample size of 6000 packets.

The relationship between PER and RSSI was investigated with a signal generator. Starting with an input power of −35 dBm, this is reduced in 1 dBm steps to −65 dBm. In each stage, 6000 packets are transmitted. For each successfully decoded WuPt, the AS3933 issues an interrupt to the MSP430. $N_{\mathrm{WuPt}\_\mathrm{Rx}}$ indicates the total number of interrupts. Then, the interrupts are logged and compared with the total number of transmitted WuPts $N_{\mathrm{WuPt}\_\mathrm{Tx}}$. To obtain a practical indication of the sensitivity of the WuRx, the PER is measured at each iteration. The PER can be calculated as presented in Equation (16).

(16) $$\mathrm{PER}=1-\frac{N_{\mathrm{WuPt}\_\mathrm{Rx}}}{N_{\mathrm{WuPt}\_\mathrm{Tx}}}$$

**Figure 19:**
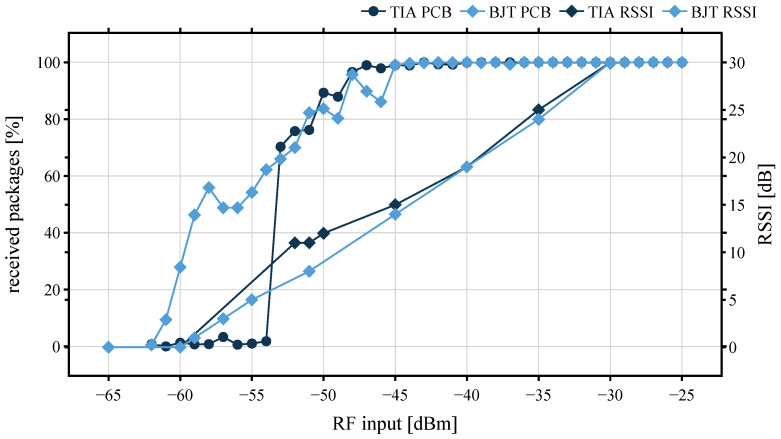
Packet error rate depending on input power.

The relationships of received WuPts against input power, as well as the RSSI value, are shown in Figure 19. With a probability of 96%, the TIA and BJT platines detect the packets at an input signal down to −48 dBm. Both architectures receive reliably up to a sensitivity of almost −50 dBm. With a packet receiving rate of around 1%, the lowest sensitivity of the BJT is −62 dBm and RSSI drops to 1, whereas the TIA can only detect packets up to an input power of −54 dBm, due to the noisy LDO of the AS3933. This shows that it is not only the sensitivity of the WuRx that matters, but the reliability of the reception rate must also be taken into account.

### 4.4. Indoor Operational Range Evaluations

**Figure 20:**
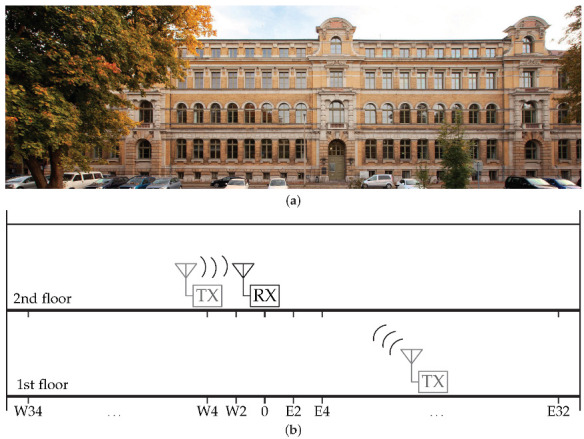
Setup of indoor range test. (**a**) Range test at the Faculty of Engineering of HTWK Leipzig (credit: Swen Reichhold/HTWK Leipzig); (**b**) Schematic side view of the measurement setup.

The performance of the WuRx is influenced by various environmental parameters. Especially indoors, various influencing factors can affect the communication between nodes. The wake-up receiver has been tested in the building of Faculty of Engineering of Leipzig University of Applied Science (Figure 20a) to ensure the reliability of the complete wake-up system when transmitting the complete WuPt with 16 bit node address. The building provides a challenging environment to explore limits of wireless communication with WuRx. Materials such as stone, glass, concrete, and steel are built in and restrict the propagation of waves.

The experimental investigation is performed in different rooms and floors. Among other things, the PER and RSSI are used as comparison values to investigate influences of masonry and furniture. Although the SAW filter at the input of the WuRx provides higher immunity to signal interference and the passive amplifier increases sensitivity, the maximum range and reliability of delivered packets are still affected.

The main receiver of the BJT and TIA boards was located on the second floor in the middle of the corridor, while the main transmitter was placed on two floors. Firstly, the same floor as the receiver was located, and, secondly, the floor underneath and the adjacent rooms. The experimental study was conducted at a room temperature of 25 ${}^{\circ }$C and a distance starting from 0 m. The transmitter was moved towards the WuRx in 4-m increments in the corridor and in 2-m increments in the rooms (see Figure 20b).

Once the WuRx boards were placed—marked in figures with (x)—the transmitter was started to send the wake-up packets. For each configuration, a result grid was created representing the packet error rate. The WuPt signals were transmitted every 50 ms with a transmission power of 11 dBm. For each grid point, the receiver counts the arriving packets and records the RSSI. The maximum distance (*d*) on the plane was 34 m with a maximum orthogonal distance of 8 m and a height difference of 0.5 m between transmitter and receiver.

**Figure 21:**
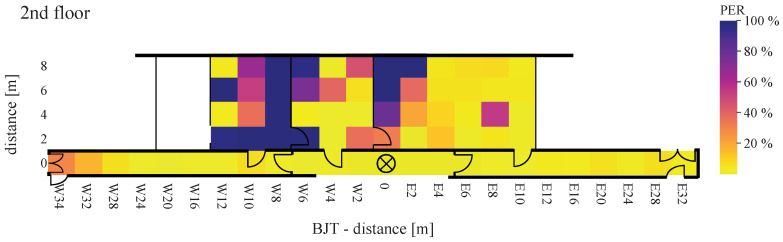
Packet error rate of BJT board with WuRx and WuTx on the same floor.

**Figure 22:**
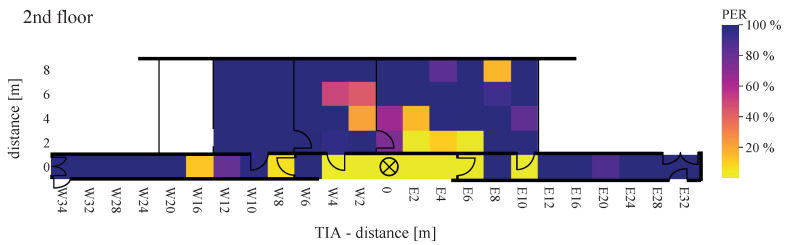
Packet error rate of TIA board with WuRx and WuTx on the same floor.

**Figure 23:**
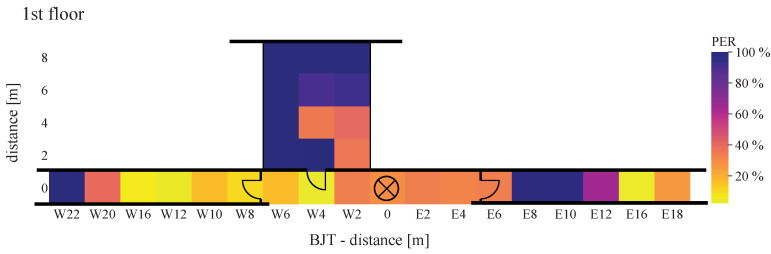
Packet error rate of BJT board with WuRx and WuTx on different floors.

**Figure 24:**
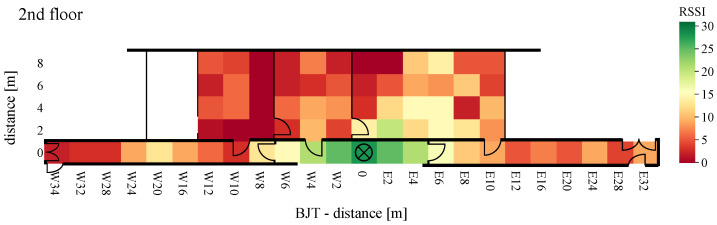
RSSI of BJT board with WuRx and WuTx on different floors.

**Figure 25:**
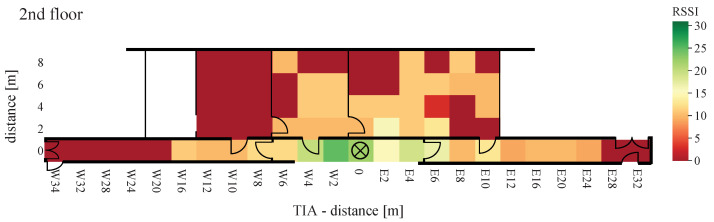
RSSI of TIA board with WuRx and WuTx on different floors.

**Figure 26:**
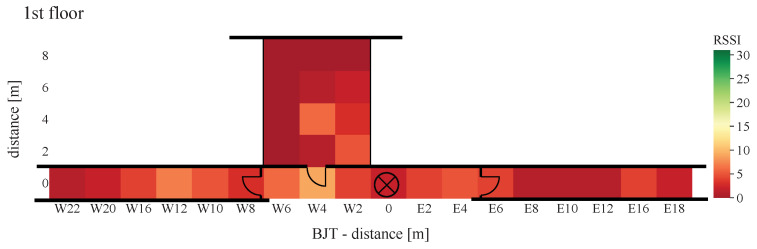
RSSI of BJT board with WuRx and WuTx on different floors. TIA implementation was not able to receive a single packet at this floor level.

Using Equation (16), Figure 21, Figure 22, Figure 23, Figure 24, Figure 25 and Figure 26 show the PER of BJT and TIA boards set up in the building.

Figure 21 and Figure 22 display the receiving and transmitting device placed in the same floor. The x-axis represents the east–west direction of each floor. The y-axis represents the north–south direction of each floor, where southwards are limited by zero. If both units—transmitter and receiver—are located on the same floor, a lower PER value for distance is displayed. This applies to BJT and TIA amplifiers.

The maximum reception rate is displayed when the devices are close to each other. Expanding the distance by placing one of the devices on another floor, the PER between WuRx with BJT amplifiers is almost higher than 10%, even at the ideal horizontal distance. By communication over floors, at a distance of 22 m in the west direction (W22) and of 18 m in the east direction (E18), the PER between WuRx with TIA amplifier at 100% means no signal is received. Additionally, with the TIA amplifier, the PER increases rapidly when the devices are placed in different rooms. Comparing the BJT and TIA amplifiers, it can be seen that the amplifier circuitry is very sensitive to vertical and horizontal distances. In ideal positions, both amplifiers provide a similar PER between WuRx of about 0%. In the indoor corridor case, reliable awakening was found up to a distance of 34 m, where awakening success was 90% or more.

At a distance greater than 20 m and placement of each equipment in different floors, with the TIA amplifier, not a single packet was received. The second comparison criterion is the RSSI provided by the AS3933. When a signal is detected, the value is set between 0 and 31. The measured signal strengths of the received WuPts are shown in Figure 24, Figure 25 and Figure 26.

The highest RSSI was achieved when both devices were on the same floor and close to each other. At a distance of more than 4 m, the RSSI dropped abruptly from 30 to below 15. The RSSI was significantly lower when the transmitters were placed in a room, similar to the PER. When both devices were positioned on different floors, the maximum signal strength did not exceed 10.

The measurements were performed under realistic, unshielded indoor conditions. Due to this, reflections, as well as interference, may be present in the ISM band, causing error modes in the passive front-end architecture of the receiver. This occurs at a certain power level (the power of the interfered at the wake-up receiver) because the power level of the coherent interferences does not vary with a Gaussian probability distribution like the receiver noise [48]. This results in a more abrupt transition from a low PER to not a single successful WuPt reception. Similarly, the attenuation due to furniture can be clearly seen in the results. No signal was received in grid points W8(2–8) to W12 in Figure 21 and Figure 24 due to the metal cabinets, which attenuates the radio signal to less than the lowest detectable power of 80 μVRMS.

## 5. Conclusions

WuRx and asynchronous communication offer significant energy savings for wireless sensor networks compared to synchronized communication protocols. Various approaches are possible to enhance the performance of WuRx, including the use of RF and LF amplifiers to increase the range of sensor nodes. As RF amplifiers, LNAs achieve sensitivities of up to −90 dBm and a data rate of several 100 kbit/s, but, on the other hand, higher latency, longer listening times, and higher power consumption. In order to achieve a sensitivity of more than −50 dBm within a power consumption lower than 10 μW, two LF amplifiers based on OA and BJT are introduced. However, the hardware of WuRx is still challenging in terms of compromise between sensitivity, data rate, latency, and low power consumption. In this work, two WuRx architectures based on LF amplifiers were investigated. In order to build a wireless sensor network for indoor localization and a multi-hop structure, the results were determined by analytical modeling, simulation, and experimental testing to compare the architectures in terms of influencing factors.

The first architecture uses for the amplification stage a BJT architecture where the second one is based on OA. Results show that the BJT architecture can be placed at a distance of 34 m with a reliability of the WuPt reception with a PER below 10%, where the other architecture presents a distance of 6 m with a PER below 10%. When communicating between rooms through masonry, a high reception rate can be expected at a distance of more than 10 m, depending on the presence and, ultimately, the amount of furniture and objects in the rooms. Communication between several floors allows a secure wireless connection at a distance of up to 12 m. The paper shows that a wireless sensor network can be built with passive and discrete components such as Schottky diodes, BJT transistor, and OA with indoor ranges of more than 30 m, with a PER of less than 10%. The limitation of the range in the rooms is mainly due to the furniture and interference with other radios such as transponder locks. The results show that LF amplifiers improve the sensitivity of the WuRx with low power requirement. However, when developing the hardware, it should be mentioned the trade-off between sensitivity, power consumption, and data rate.

## Figures and Tables

**Table 1:** Comparison of data rate and wake-up packets.

Reference	[25]	[27]	[29]	[30]	[31]	[32]	[33]	This Work
Frequency (MHz)	868	868	868	868	868	433	433	868
Sensitivity (dBm)	−53	−51	−71	−90	−70	−63	−63	−62
Voltage amplification	none	VM	LNA	LNA	OA	BJT	BJT	OA and BJT
Listening mode	AO ^1^	AO	DC ^2^	DC	AO	AO	AO	AO
Data rate (kbit/s)	2.7	1.0	250	128	0.04	1.0	1.0	1.2
Current requirement transmitter ^3^ (μAs)	148	400	165	803	10,000	400	400	345

^1^ Always on. ^2^ Duty-cycle. ^3^ Estimate for 16-bit address and 25 mA supply current.

**Table 2:** Summary of component values for the wake-up receiver.

Components	Parameters	Values
Antenna ANT-868-CW	Center Frequency	868 MHz
	Impedance	50 Ω
	Peak Gain	−2.3 dBi
	Bandwidth	30 MHz
SPIRIT1 SPSGRFC-868	Supply Current TX Mode	21 mA
	Supply Current RX Mode	9 mA
SAW Filter B39871B	Center Frequency	869 MHz
	Maximum Insertion Attenuation	2.5 dB
	Usable Pass band	2 MHz
Schottky Diodes SMS7630	Voltage Sensitivity	40 mV/μ
	Video Resistance	5 kΩ
BFP405 NPN RF Bipolar Transistor	Maximum Gain	23 dB
	Minimum Noise Figure	1.25 dB
	Collector Current	1.3 μA
MIC861 Operational Amplifier	Supply Current	4.2 μA
	Gain Bandwidth Product	350 kHz
AS3933 LF Wake-Up-Chip	Supply Current Scanning Mode	3.1 μA
	Supply Current Preamble Detection	12 μA
MSP430 Microcontroller	Supply Current Standby	860 nA
	Supply Current Active	2 mA
	System Clock	8 MHz

**Table 3:** Structure of the WuPt with length.

Segment	Length	Duration (ms)
Carrier burst	32 pulses	8.3
Preamble	6.5 periods	5.6
Address	16 bits	13.8

**Table 4:** Energy consumption of BJT and TIA amplifiers.

Mode	TIA	BJT
Listening * [μJ]	28.35	25.05
Receiving [μJ]	1.52	1.28
Transmission [mJ]	1.91	1.91

^*^t_listen_ = 1 s.

## Abbreviations

The following abbreviations are used in this manuscript:

AGC	automatic gain control
ASK	amplitude shift keying
BJT	bipolar junction transistor
CMOS	complementary metal-oxide semiconductor
COTS	commercial of the shelf
FSK	frequency shift keying
GBWP	gain bandwidth product
IoT	Internet of Things
ITU	International Telecommunication Union
LED	light-emitting diode
LF	low frequency
LNA	low noise amplifier
MCU	main microcontroller unit
OA	operational amplifier
OOK	on-off-keying
PCB	printed circuit board
PER	package error rate
RF	radio frequency
RSSI	received signal strength indicator
SAW	surface acoustic wave
SoA	State of the Art
TIA	transimpedance amplifier
TSS	tangential signal sensitivity
VGA	variable gain amplifier
WSN	wireless sensor network
WuPt	wake-up packet
WuRx	wake-up receiver
WuTx	wake-up transmitter
